# Drug Release Analysis and Optimization for Drug-Eluting Stents

**DOI:** 10.1155/2013/827839

**Published:** 2013-12-29

**Authors:** Hongxia Li, Yihao Zhang, Bao Zhu, Jinying Wu, Xicheng Wang

**Affiliations:** ^1^State Key Laboratory of Structural Analysis for Industrial Equipment, Department of Engineering Mechanics, Dalian University of Technology, Dalian 116024, China; ^2^Surface Engineering Laboratory, School of Materials Science and Engineering, Dalian University of Technology, Dalian 116024, China

## Abstract

The drug release analysis and optimization for drug-eluting stents in the arterial wall are studied, which involves mechanics, fluid dynamics, and mass transfer processes and design optimization. The Finite Element Method (FEM) is used to analyze the process of drug release in the vessels for drug-eluting stents (DES). Kriging surrogate model is used to build an approximate function relationship between the drug distribution and the coating parameters, replacing the expensive FEM reanalysis of drug release for DES in the optimization process. The diffusion coefficients and the coating thickness are selected as design variables. An adaptive optimization approach based on kriging surrogate model is proposed to optimize the lifetime of the drug in artery wall. The adaptive process is implemented by an infilling sampling criterion named Expected Improvement (EI), which is used to balance local and global search and tends to find the global optimal design. The effect of coating diffusivity and thickness on the drug release process for a typical DES is analyzed by means of FEM. An implementation of the optimization method for the drug release is then discussed. The results demonstrate that the optimized design can efficiently improve the efficacy of drug deposition and penetration into the arterial walls.

## 1. Introduction

Cardiocerebrovascular disease is a serious threat to human health. There are three main treatments for vascular diseases: surgery, coronary angioplasty, and coronary stenting. Coronary stenting is minimally invasive catheter-based interventions. Compared to surgery, the coronary stenting is less invasive, so postoperative recovery is quick. Compared to coronary angioplasty, it can avoid restenosis, efficiently. So coronary stenting has been widely applied to clinical; so far coronary stenting technique has become the most promising treatment for coronary artery diseases; however, the arterial wall damage and restenosis caused by stent have not been completely resolved. This is the main reason that development of stenting is hampered. Fortunately, the coronary stent can carry the drug through drug eluting. The drug-eluting stents (DES) could provide the local high concentration of the drug by local drug delivery system and minimize the systemic side effects. Thereby, the generation of thrombus is suppressed, and the risk of restenosis is reduced [1]. Therefore, the DES is a revolutionary technology for stenosis disease in the clinical treatment. However, the drug release in the blood and drug concentration gradient in the blood vessels are complex fluid dynamics problems. How to control the drug release in the blood and drug concentration gradient in the blood vessels is a challenging task. So dosing and extending the efficacy period are very important.

A few works about DES were reported, Yang and Burt [1] explored several factors that impact the drug release of DES, such as physiological transport forces, drug physicochemical properties, local biological tissue properties, and stent design. Pontrelli and de Monte [2] proposed a novel computational approach and studied the impact of tissue properties, local hemodynamics, and stent design for the drug release of DES based on a consistent mathematical model. The previous work for DES mainly researched the drug release of DES but did not propose how to extend the efficacy of DES. Drug release process is very complex, and the drug concentration is nonuniform. The relative function of drug concentration and the factors that affect drug release cannot be described by an explicit expression. So it is very difficult to propose how to extend the efficacy of DES. Fortunately, kriging surrogate model can be used to establish the approximate functional relationship between drug concentration and the factor of drug release, effectively. Based on the kriging surrogate model, we can optimize the design of the DES and extend the efficacy of DES easily.

The Finite Element Method (FEM) is here used to analyze the process of drug release in the vessels for DES, in which the microstructure of tissue (anisotropic diffusion of the drug, porosity, and retention of the drug protein) and the macrostructure of tissue (thrombus/blood clots) are considered. Based on the FEM analysis, an optimization approach combined with Expected Improvement (EI) function [3] which is based on kriging surrogate model [4] is used to extend the lifetime of the drug in artery wall. The diffusion coefficients and the coating thickness are selected as design variables. The Latin Hypercube Sampling Method is used to obtain sample points for the initial model established by kriging surrogate model method; EI function is used to balance local and global search and tends to find the global optimal design.

## 2. Methods

**Figure 1:**
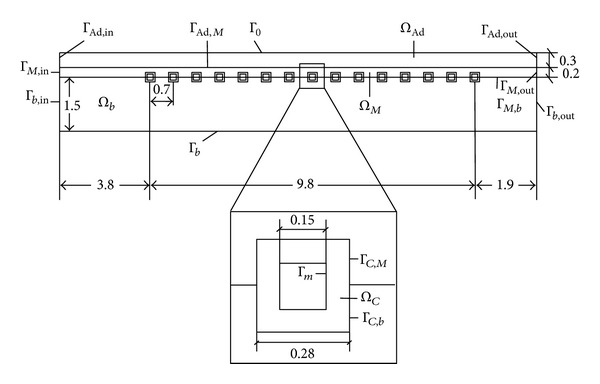
2D simplified model.

The Finite Element Method (FEM) is used to analyze the process of drug release in the vessels for Drug Eluting Stents (DES). As shown in Figure 1, a simplified axisymmetric model [5] for (DES) is adopted for quantitative analysis, which was proposed to research the drug release of the DES by Mongrain et al. [6]. As the intima and media layers have similar property and the stent is usually embedded in media layer, the arterial wall is modeling with two layers (intima and media layer and adventitia layer).

As shown in Figure 1, *Ω*_*M*_, *Ω*_Ad_, *Ω*_*C*_, and *Ω*_*b*_ are the intima and media layer, the adventitia layer, the coating, and the blood, respectively. Γ_*s*_ is the symmetry axis of the blood, Γ_*b*,in_ is the inflow boundary, Γ_*b*,out_ is the outflow boundary, Γ_*m*_ is the interface of coating and stent, and Γ_*M*,in_ and Γ_*M*,out_ are the artery boundaries of inflow and outflow for intima and media layer, respectively. Γ_Ad,in_ and Γ_Ad,out_ are the artery boundaries of inflow and outflow for adventitia layer. Γ_0_ is the interface of arterial wall and trophoblast tube; Γ_*C*,*b*_, Γ_*C*,*M*_, Γ_*M*,*b*_, and Γ_Ad,*M*_ are the interfaces of blood, artery, and coating, respectively.

At initial time (*t* = 0), the drug was assumed completely dissolved in the coating and the concentration is uniform. The coating is modeled as a homogeneous isotropic porous media, and plasma cannot penetrate the coating. The control equation, boundary condition and initial time in the coating can be found in [5].

The arterial wall is also modeling as porous media. Because the inner and outer walls in the presence of physiological arterial pressure will lead to plasma flow, mass transfer was under diffusion and convection equations [7]. The convection effect is only acting on the drug dissolved in the blood, according to the report by Creel et al. [8], without considering transient absorption effect of the drug in artery, and the drug concentration in artery is *c*′ = *c*/*kε*, in which *c* is the average drug concentration in arterial tissue and *k* is the partition coefficient in arterial wall, and *ε* is the arterial porosity. On the interface of artery and coating, the boundary conditions are the same as reference [5].

The control equation of blood flow is the general convection diffusion equation. The control equation and the boundary condition on the interface of artery and coating can be found in reference [5].

First of all, considering the plasma flow in the arterial wall, the plasma flow velocity in arterial wall, and the pressure difference between inside and outside surfaces determined by the Darcy law, the blood in artery is modeled as incompressible Newtonian Equation. As the drug release in the blood does not affect blood flow, the velocity field can be obtained, and then, with the velocity field as given conditions, the drug concentration distribution at different times can be obtained [5].

The material properties are the same in reference [5]. The blood velocity distribution at the entrance is as parabolic shape. The maximum speed is 14.0 cm/s. The drug diffusion in coating, blood, and adventitia layer is isotropic. The drug diffusion in intima and media layer is anisotropic. The coefficients of control equations are the same as reference [5].

**Figure 2:**
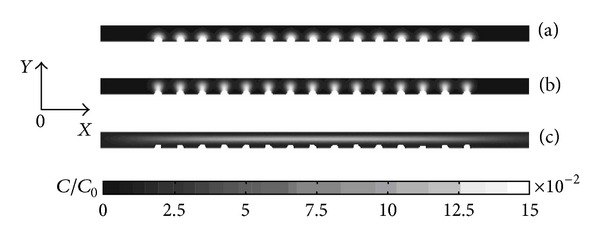
Drug concentration of the artery wall with different models. (a) Isotropic nonporous medium, (b) isotropic porous media, and (c) anisotropic porous media.

Figure 2 shows the difference between isotropic and anisotropic diffusion coefficients. Drug distribution is more uniform in the *x* direction for anisotropic diffusion coefficients than isotropic diffusion coefficients because it is easier in the horizontal direction (the axial direction) than the vertical diffusion direction (radial direction) and seepage flow of plasma in porous media. The convection effect of *y* direction promotes the diffusion of the drug.

**Figure 3:**
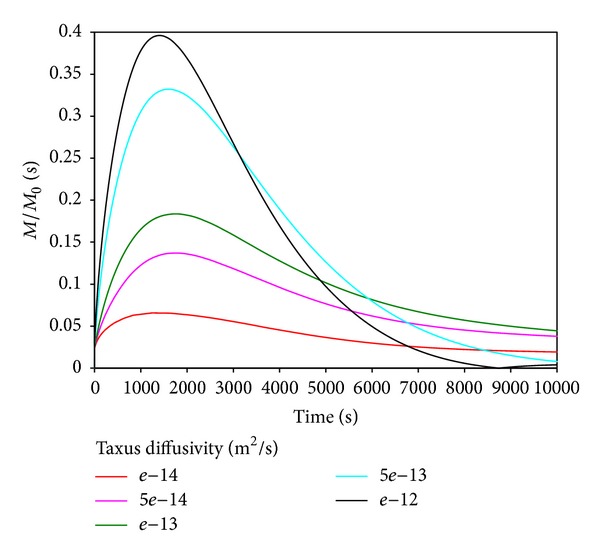
Influence of coating diffusivity on drug concentration into the artery wall.

Figure 3 shows the drug concentration versus time with different diffusion coefficients in the coating. It is clear that drug concentration reached the peak in arterial wall, and then it decreased. It is reasonable; because drug will diffuse into artery, gradually by the difference of drug concentration in the coating and artery, and due to the elution effect, the drug concentration decreased outside of the artery wall. The elution effect is not significant at initial moment because of the low drug concentration in the artery wall. When the drug reaches certain concentration in artery wall and drug release to a certain degree in coating, the elution effect will be quite significant. As the coating cannot continue to provide drugs continuously, the drug concentration will decrease.

As the diffusion coefficient increases, the drug concentration in the artery wall reaches a higher peak with a faster growth. The decay rate of the peak is not proportional to the diffusion coefficient, so the trend of the change of drug concentration is not linear relationship with diffusion coefficient. This conclusion is important, because if we want a longer time drug concentration in the artery wall, it should not increase or decrease the diffusion coefficients of the coating, simply. Therefore, it appears optimal to find an appropriate diffusion coefficient to make the longest time with certain drug concentration. That provides a theoretical basis for DES coating optimization.

**Figure 4:**
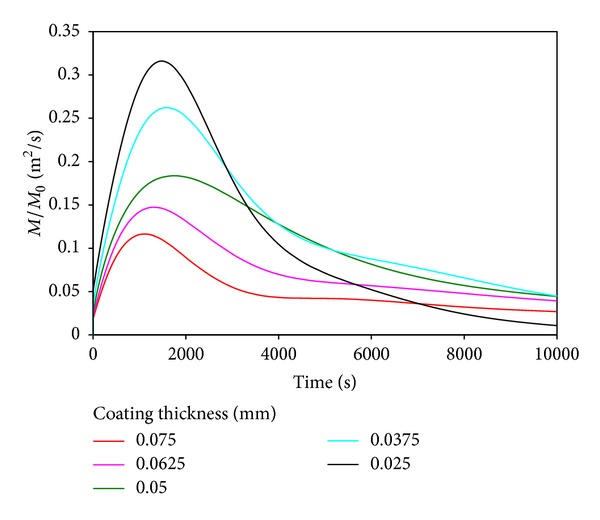
Influence of coating thickness on drug concentration into the artery wall.

Figure 4 shows drug concentration versus time with different thicknesses of coating in the artery wall. It is clear that drug concentration reached the peak in arterial wall, and then it decreased. It is because drug will diffuse into artery, gradually by the difference of drug concentration in the coating and artery, due to the elution effect, the drug concentration decreased outside of the artery wall. The elution effect is not significant at initial moment because of the low drug concentration in the artery wall. When the drug reaches certain concentration in artery wall and drug release a certain degree in coating, the elution effect will be quite significant. As the coating cannot continue to provide drugs continuously, the drug concentration will decrease.

The initial concentration of drugs in the coating shifted to a higher level with the decrease of the coating thickness. It is clear that the drug concentration in the artery wall reaches a higher peak with a faster growth. The duration of the drug in the artery wall is not inversely proportional to the coating thickness. So the trend of the change of drug concentration is not linear relationship with the coating thickness. Therefore, it appears optimal to find an appropriate coating thickness.

The two important factors for the distribution of drugs in artery wall are diffusion coefficients and the coating thickness. Therefore, diffusion coefficients and the coating thickness can be chosen as the design variables. An optimization approach based on kriging surrogate model is used to optimize the lifetime of the drug in artery wall. The kriging model was used to build an approximate function relationship between the objective function and design variables (the diffusion coefficients in coating and the thickness of coating), thereby replacing the expensive FEM reanalysis of the objective function value during the optimization. The optimization iterations are based on the approximate relationship for reducing the high computational cost. An adaptive optimization method based on the kriging surrogate model with Latin Hypercube Sampling (LHS) strategy [9] was used to improve the effect of the drug release of DES. The adaptive process was implemented by the EI function [10], which can balance local and global searches and tends to find the global optimal design. The FEM was used to analyze the drug release of DES in stented artery.

The evaluation standard of effect of the drug release on the stent is Mean Residence Time (MRT) [2]: the equation is as follows

(1) MRTn=max⁡{t ∣ ∭wallcwall(x,y,z,t)dx dy dz     ≥n100∭polymerc0(x,y,z,0)dx dy dz}.

MRT is an important indicator for judging the effect of the drug-eluting stent. In this paper, MRT_10_ is the objective function, assuming that the initial drug concentration is constant in the coating. The diffusion coefficients in the artery wall are constant, and the shape of the stent remains unchanged. For the range of design variables, the range of the diffusion coefficients is 10^−12^ m^2^/s~10^−14 ^m^2^/s; range of the coating thickness is 0.023 mm~0.075 mm. The sample points and the corresponding response are listed in Table 1.

## 3. Results and Discussion

**Figure 5:**
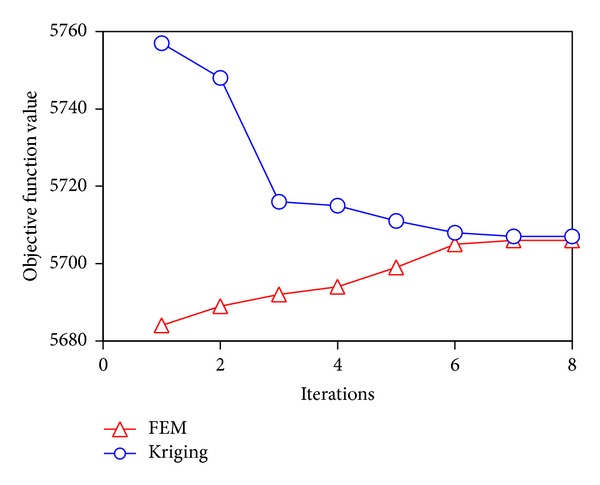
Iterative optimized process.

As shown in Figure 5, the optimization results have fast convergence. The study indicates that when the diffusion coefficient equals 2.5 × 10^−13^ m^2^/s and the coating thickness equals 0.05 mm, the drug duration time reaches its maximum under MRT_10_.

**Figure 6:**
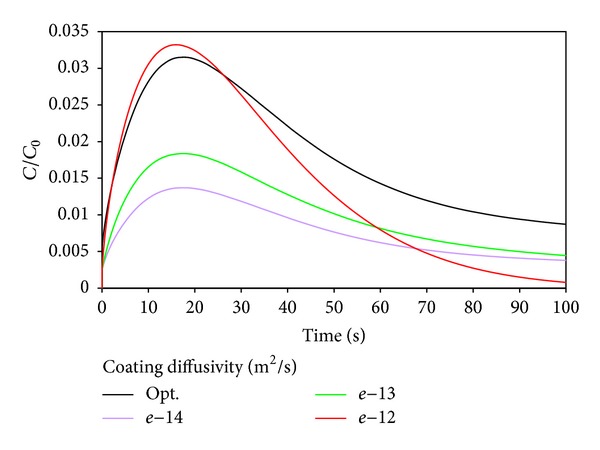
Influence of coating diffusivity on drug concentration into the artery wall.

**Figure 7:**
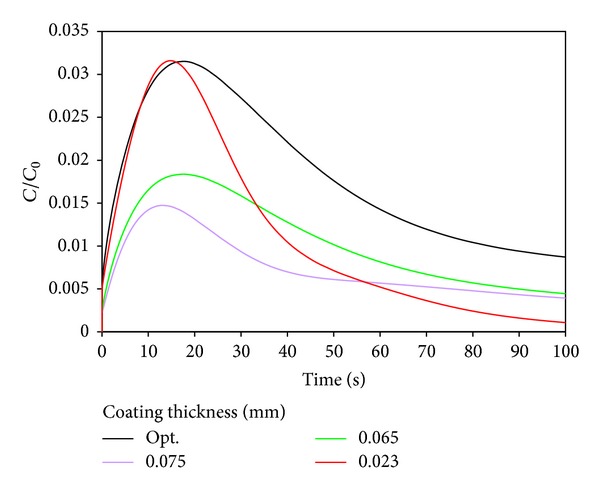
Influence of coating thickness on drug concentration into the artery wall.

Another judge standard is the ratio of mean concentration and initial concentration in the media layer. So the objective function is selected as the drug duration time when C_*m*_/C_0_ ≥ 1%, and the design variables are also diffusion coefficients and the coating thickness. As shown in Figures 6 and 7, the optimized objective function reached 85 h, and the drug duration time is increased by 41 h compared to initial design. The optimal diffusion coefficient is 3.2 × 10^−13^ m^2^/s, and optimal coating thickness is 0.05 mm. It will release the drug into arterial wall faster by increasing the diffusion coefficient and reducing the coating thickness. However, when the drug concentration is too high, concentration difference will lead to lower drug diffusion. At the same time, the drug will diffuse into the outer layer because of the effect of convection caused by the seepage flow in arterial wall. This is the reason why the concentration decreased rapidly from the peak. So our results are reasonable.

## 4. Conclusions

This paper studies the relationship between the properties of the coating and the drug distribution in the arterial wall. The study indicates that diffusion coefficients and the coating thickness are two important factors of the distribution of drugs in artery wall. An optimization approach based on kriging surrogate model is used to optimize the lifetime of the drug in artery wall. The diffusion coefficients and the coating thickness are selected as design variables. The results demonstrate that the optimized design can efficiently control the release of drug in the blood and drug concentration gradient in the blood vessels.

## Figures and Tables

**Table 1:** Samples and responses.

Samples	Taxus diffusivity (m^2^/s)	Coating thickness (mm)	Response (s)
1	4.82∗10^−13^	0.057	4238
2	1.07∗10^−13^	0.052	3658
3	9.45∗10^−13^	0.073	3670
4	8.68∗10^−13^	0.042	3416
5	1.53∗10^−13^	0.065	2928
6	8.80∗10^−13^	0.031	3078
7	3.84∗10^−13^	0.027	3940
8	6.55∗10^−13^	0.059	4104
9	8.07∗10^−13^	0.072	3782
10	7.44∗10^−13^	0.032	3112
11	6.84∗10^−13^	0.065	3862
12	5.55∗10^−13^	0.046	4604
13	2.37∗10^−13^	0.038	4862
14	5.87∗10^−13^	0.041	3984
15	3.54∗10^−13^	0.062	4120
